# Neutrophil gelatinase associated lipocalin (NGAL) is elevated in type 2 diabetics with carotid artery stenosis and reduced under metformin treatment

**DOI:** 10.1186/s12933-017-0579-6

**Published:** 2017-08-08

**Authors:** W. Eilenberg, S. Stojkovic, A. Piechota-Polanczyk, A. Kaider, N. Kozakowski, W. J. Weninger, J. Nanobachvili, J. Wojta, I. Huk, S. Demyanets, C. Neumayer

**Affiliations:** 1 Department of Surgery, Division of Vascular Surgery, Medical University of Vienna, Währinger Gürtel 18-20, 1090 Vienna, Austria; 2 Department of Internal Medicine II, Division of Cardiology, Medical University of Vienna, Vienna, Austria; 3 Department of Medical Biotechnology, Faculty of Biochemistry, Biophysics and Biotechnology, Jagiellonian University in Krakow, Krakow, Poland; 4 Center for Medical Statistics, Informatics, and Intelligent Systems, Medical University of Vienna, Vienna, Austria; 5 Department of Pathology, Medical University of Vienna, Vienna, Austria; 6 Center of Anatomy and Cell Biology, Medical University of Vienna, Vienna, Austria; 7 Core Facilities, Medical University of Vienna, Vienna, Austria; 8 Department of Laboratory Medicine, Medical University of Vienna, Vienna, Austria

**Keywords:** NGAL, Lipocalin, Atherosclerosis, Carotid artery stenosis, Inflammation, Type II diabetes, Metformin

## Abstract

**Background:**

Neutrophil gelatinase-associated lipocalin (NGAL), an acute phase protein released by neutrophils, has been described as biomarker of inflammatory states. Type 2 diabetes mellitus (T2DM) is characterized by increased inflammation and an elevated risk for embolization of carotid artery stenosis (CAS). We aimed to explore the role of NGAL systemically and in plaques of diabetics undergoing carotid endarterectomy. Moreover, the potential anti-inflammatory effect of metformin on NGAL was addressed in diabetics.

**Methods:**

Serum NGAL and matrix metalloproteinase (MMP)-9/NGAL levels were measured in 136 patients (67 with T2DM vs. 69 non-diabetics) by specific ELISA. Endarterectomy samples were graded histologically according to the American Heart Association´s classification. NGAL mRNA expression was detected using RealTime-PCR in carotid endarterectomy specimens.

**Results:**

Serum NGAL [median 107.4 ng/ml (quartiles: 75.2–145.0) vs. 64.4 (50.4 –81.3), p < 0.0001] and MMP-9/NGAL [41.5 ng/ml (20.8–63.9) vs. 27.6 (16.0–42.4), p = 0.017] were significantly elevated in diabetics compared to non-diabetics, as were leukocytes, neutrophils, C-reactive protein and fibrinogen (all p < 0.05). In patients with symptomatic and asymptomatic CAS diabetics had higher NGAL levels compared to non-diabetics [128.8 ng/ml (100.8–195.6) vs. 64.8 (48.9–82.2] and [101.6 ng/ml (70.1–125.3) vs. 63.8 (51.0–81.3), respectively, both p < 0.0001]. Presence of T2DM and type VI plaques (with surface defect, hemorrhage or thrombus) had a profound impact on NGAL levels (both p < 0.01) in multiple linear regression analysis. NGAL mRNA was detectable in 95% of analyzed carotid artery lesions of diabetics compared to 5% of non-diabetics (p < 0.0001). Accordingly, cerebral embolization was more frequent in diabetics (52.2% vs. 29%, p = 0.006). Metformin treatment was associated with decreased NGAL [60.7 ng/ml (51.9–69.2) vs. 121.7 (103.7–169.9), p < 0.0001] and MMP-9/NGAL [20.8 ng/ml (12.1–26.5) vs. 53.7 (27.4–73.4), p = 0.007] in diabetics and reduced leukocyte infiltration in carotid lesions of diabetics.

**Conclusions:**

Higher NGAL levels in serum and plaques are associated with T2DM in patients with CAS. Metformin significantly reduced the inflammatory burden including NGAL in diabetics. Early treatment of these patients may be recommended, as elevated NGAL levels were linked with vulnerable plaques prone for embolization.

## Introduction

Type II diabetes mellitus (T2DM) is one of the largest epidemics in the world. In 2015 about 420 million people were suffering from T2DM worldwide, and it has been estimated that the number will increase to more than 640 million by 2040 [1]. Since the introduction of the “Framingham study” in 1979 the increased risk for cardiovascular events in patients with T2DM is well known [2]. Their incidence for ischemic strokes, of which about 30% are related to carotid artery stenosis (CAS), is 2.5–3.5 times higher compared to non-diabetics [3, 4]. Although good glycemic control is able to reduce the risk for cardiac events, the risk for ischemic stroke remains unaltered [4]. Consequently, this may cause persistent functional impairment or even death in numerous patients.

Multiple pathophysiological pathways have been made responsible for the development of atherosclerosis promoting vascular inflammation and thrombosis [5–12]. Patients with T2DM are especially prone to inflammatory processes, which lead among others to endothelial dysfunction [7, 13, 14]. Pro-inflammatory cytokines [such as interleukins (IL-1, IL-6), and tumour necrosis factor (TNF)-α] as well as chemokines and adhesion molecules have been shown to contribute to vascular complications in T2DM [13, 15]. The enhanced inflammatory state in type 2 diabetics was also documented by increased macrophage invasion in carotid plaques and elevated matrix metalloproteinase (MMP)-9 levels [16–19]. MMP-9 plays a crucial role in plaque instability as it is able to degrade the extracellular matrix and in particular collagen [20, 21].

Neutrophil gelatinase-associated lipocalin (NGAL) or lipocalin 2, which is mainly released from granules of activated neutrophils, was recognized as an acute phase protein [22]. Increased NGAL levels have been reported in atherosclerosis and linked to inflammatory processes [23–30]. NGAL is able to bind to MMP-9 and to form a dimeric MMP-9/NGAL complex, which prolongs the deleterious effects of MMP-9 on the arterial wall, as MMP-9 degradation is thereby prevented [31]. Previous works have shown that NGAL is expressed by macrophages, smooth muscle cells and endothelial cells in human carotid plaques [23, 25]. In addition, we demonstrated that NGAL up-regulated the production of pro-inflammatory cytokines IL-6, IL-8, and monocyte chemoattractant protein (MCP)-1 in these cells in vitro [23]. Moreover, NGAL may serve as potential circulating biomarker of plaque vulnerability in patients with CAS, as was shown by our group recently [24].

The aim of our present study was to investigate, whether serum NGAL and MMP-9/NGAL levels were elevated in type 2 diabetics with CAS in comparison to non-diabetics, and whether NGAL levels could reflect the inflammatory state, defined clinically and histologically, of these patients. Furthermore, we wanted to know if neurological events correlated to NGAL levels. Moreover, NGAL mRNA expression in carotid plaques of diabetics vs. non-diabetics was analyzed. In addition, we looked at NGAL levels under treatment of metformin in diabetic patients with CAS.

## Materials and methods

### Patient’s selection

One hundred and thirty-six patients with high grade CAS treated with endarterectomies at the Medical University of Vienna, Austria, were included in the study. Patients with acute or chronic infections, autoimmune or neoplastic diseases were excluded. All subjects were Caucasian. The study has been reviewed and approved by the Ethic Committee of the Medical University of Vienna (EK 269/2009), and all participants gave informed consent.

The patients’ cohort comprised 67 patients with T2DM and 69 non-diabetics. The diagnosis of T2DM was based on the criteria established by the American Diabetes Association in 2010 and the WHO criteria of 2011 [32]. Patients with glycated hemoglobin (HbA_1c_) >6.5%, fasting plasma glucose ≥7 mmol/l or 2 h postload plasma glucose ≥11.1 mmol/l were regarded as type 2 diabetics [32, 33]. Anti-diabetic treatment comprised metformin, insulin or other anti-diabetic drugs.

Asymptomatic patients with high grade CAS and/or vulnerable, high-risk plaques according to ultrasound examination have been operated as well as symptomatic patients having had at least one neurological event directly associated with CAS within the last 6 months [34]. Coronary artery disease (CAD) and arterial hypertension was defined according to the American Heart Association (AHA) [35]. Preoperative duplex sonography was used to classify carotid plaques according to echogenicity into “soft”, calcified/hard and mixed lesions. Furthermore, extent of CAS (in percentage) and blood velocity (in m/sec) was recorded.

### Carotid endarterectomy

Carotid endarterectomy was performed under general anesthesia via the “non-touch” technique. Protective hypertension was used or shunting if oxygen saturation decreased intraoperatively by more than one-third. Systemic heparinisation (unfractionated, adjusted to body weight and renal function) was administered at the beginning of the operation to prevent from embolization. Endarterectomy was performed via a linear arteriotomy and the plaque removed for histological and biochemical evaluation. Distal intimal tacking sutures were applied when necessary and routine patching was performed.

### Blood sampling

Blood was taken from a peripheral vein prior to surgery. All blood samples were centrifuged at 3000 rpm at 4 °C for 15 min and serum and plasma were stored in aliquots at −80 °C for further analysis.

### NGAL and MMP-9/NGAL measurement

Neutrophil gelatinase-associated lipocalin and MMP-9/NGAL levels were measured by specific enzyme-linked immunosorbent assays (ELISA, both from R&D Systems; catalog number DLCN20 and DM9L20, respectively) in serum of 136 patients. Investigators blinded to clinical and demographic data performed assays.

### Total RNA purification and cDNA preparation

Atherosclerotic tissue (i.e. endarterectomized plaques) from 39 patients was available for total RNA purification and stored at −80 °C. Frozen samples were homogenized using a ball mill (Retsch, Haan, Germany), and mRNA were isolated using RNeasy^®^ Mini Kit (Quiagen, Valencia, CA, USA). Total RNA content was measured using NanoDrop (Thermo Scientific, Barrington, IL, USA). Reverse transcription were performed using GoScript™ reverse transcription system (Promega, Madison, WI, USA).

### Real-time polymerase chain reaction

Real-time-PCR was performed using LightCycler Taq-Man Master (Roche, Basel, Switzerland) according to the manufacturer’s instructions. Primers (forward (fwd) and reverse (rev)) were designed using the Roche Universal ProbeLibrary Assay Design Centre (http://www.universalprobelibrary.com/): glycerinaldehyd-3-phosphat-dehydrogenase (GAPDH) (fwd: AGCCACATCGCTCAGACAC, rev: GCCCAATACGACCAAATCC, UPLprobe #60; Amplicon Size [bp] 66)—NGAL (fwd: CAGGACTCCACCTCAGACCT; rev: CCAGGCCTACCACATACCAC, UPLprobe #84; Amplicon Size [bp] 109). The amplification conditions consisted of an initial incubation at 95 °C for 10 min, followed by 45 cycles of 95 °C for 10 s, 63 °C for 20 s and 72 °C for 6 s and a final cooling to 40 °C. Data were analyzed using Light-Cycler Software Version 3.5 (Roche).

### Histology

Histological classification of endarterectomy specimens was performed according to the AHA [36]. Plaques were assorted into type IV plaques (confluent extracellular lipid core), type V (fibroatheroma), type VI (complex plaque with possible surface defect, hemorrhage, or thrombus), type VII (calcified plaques), and type VIII plaque (dominated fibrous tissue). Paraffin-embedded specimen were cut in 4 µm thick sections and stained with hematoxylin and eosins (H&E), Elastica van Gieson and alcian blue. The analyzing pathologist was blinded to clinical and demographic data. Descriptive analysis of histological specimen was added with special reference to leukocyte infiltration.

### Statistical analysis

Median (quartile) values were given to describe continuous variables; absolute numbers and percentages are used to describe categorical variables. Differences between patient groups with respect to NGAL and MMP-9/NGAL levels, respectively, were tested by the two-sample t test. In case of more than two groups, analysis of variance models were performed, and the Tukey method was applied to correct for multiple comparisons. Differences in continuous variables of diabetes patients compared to non-diabetes patients were tested using the two-sample t test, non-normally distributed variables were compared by the Wilcoxon rank sum test. Categorical variables were compared by the Chi square test. Correlations of continuous variables were characterized using the Spearman correlation coefficient.

Univariate and multiple linear regression models were performed to evaluate the unadjusted and adjusted impact of the variables T2DM, creatinine, neutrophils, body mass index (BMI), plaque ultrasound, histological classification of the plaques and symptomatic presentation of CAS on the NGAL and MMP-9/NGAL levels. Log-transformed values of neutrophils and MMP-9/NGAL and rank-transformed values of creatinine were used for statistical analyses due to their skewed distributions. To evaluate the influence of anti-diabetic treatment on NGAL and MMP-9/NGAL levels in peripheral blood, diabetic patients were compared with respect to the three types of treatment: insulin, metformin, and other anti-diabetic treatment. Furthermore, univariate and multiple linear regression models were applied on data of diabetic patients to evaluate the unadjusted impact of the treatments on NGAL and MMP-9/NGAL and adjusted for HbA1c and neutrophils. All p values are results of two-sided tests and p values <0.05 were considered as indicating statistical significance. The SAS software version 9.4 (SAS Institute Inc. 2002–2012; Cary, NC, USA) was used for statistical analyses.

## Results

### Characteristics of study population

Detailed information of the study population is given in Table 1. Diabetic and non-diabetic patients were comparable in sex, age and BMI. Cardiovascular risk factors such as smoking and hypertension were also equally distributed in both groups. In contrast, diabetics had an increased prevalence of CAD (39.4% vs. 22.1%, p = 0.030) and cerebral infarctions (52.2% vs. 29.0%, p = 0.006). Some of them exhibited silent infarctions. As a consequence 44.8% of diabetics were assessed as “symptomatic” patients having experienced transient ischemic attacks (TIA) and strokes within the last 6 months vs. 33.3% in non-diabetics (p = 0.171).

**Table 1:** Patient characteristics

	Diabetic patients (n = 67)	Non-diabetic patients (n = 69)	p
Age (years), median (quartiles)	72.0 (64.0–75.0)	69.0 (64.0–74.0)	0.288*
Sex (female) (%)	40.3	49.3	0.293**
Body mass index (kg/m^2^), median (quartiles)	28.1 (24.7–30.5)	27.1 (24.2–29.1)	0.234*
Smoker (%)	39.4	36.8	0.754**
Hypertension (%)	85.1	87.0	0.752**^+^
Hyperlipidemia (%)	77.6	43.5	<0.0001**
CAD (%)	39.4	22.1	0.030**
Cerebral infarction (%)	52.2	29.0	0.006**
Symptomatic (TIA/Stroke) (%)	44.8	33.3	0.171**
T-ASS (%)	71.6	85.5	0.048**
ACE-inhibitor (%)	38.5	35.8	0.754**
Statins (%)	68.7	89.9	0.002**
Creatinine (mg/dl), median (quartiles)	0.98 (0.86–1.19)	0.88 (0.80–1.03)	0.023^++^
Total cholesterol (mg/dl), median (quartiles)	159.0 (132.5–188.5)	170.5 (151.5–205.5)	0.037^++^
LDL (mg/dl), median (quartiles)	83.5 (62.0–105.2)	83.6 (70.2–117.8)	0.351^++^
HDL (mg/dl), median (quartiles)	45.0 (37.0–55.0)	52.0 (43.0–63.0)	0.010*
Triglyceride (mg/dl), median (quartiles)	125.0 (95.5–211.5)	137.0 (95.5–179.0)	0.771^++^
Leukocytes (G/l), median (quartiles)	8.39 (7.10–10.03)	7.16 (6.38–9.02)	0.030*
Neutrophils (G/l), median (quartiles)	5.8 (4.7–6.8)	4.6 (3.9–6.1)	0.002^++^
CRP (mg/dl), median (quartiles)	0.68 (0.27–2.06)	0.43 (0.17–1.01)	0.014^++^
Fibrinogen (mg/dl), median (quartiles)	422.0 (382.0–490.0)	390.5 (330.5–438.5)	0.003^++^
HbA_1c_ (%), median (quartiles)	6.8 (6.0–7.4)	5.8 (5.5–6.0)	<0.0001*

Data are presented as frequencies or median (quartiles)

* t test

** Chi square test

^++^ Wilcoxon rank sum test

*CAD* coronary artery disease, *TIA* trans-ischaemic attack, *T-ASS* acetyl salicylic acid, *ACE* angiotensin-converting enzyme, *LDL* low-density lipoprotein, *HDL* high density lipoprotein, *CRP* C-reactive protein, *HbA*_*1c*_ glycated hemoglobin

Statins (89.9% vs. 68.7%, p = 0.002) and acetyl salicylic acid (85.5% vs. 71.6%, p = 0.048) were taken in a higher percentage in non-diabetic vs. diabetic patients. The prevalence of hyperlipidemia was higher in diabetics (77.6% vs. 43.5%, p < 0.0001). In contrast, high-density lipoproteins (HDL) (52 mg/dl vs. 45 mg/dl, p = 0.01) and total cholesterol (170.5 mg/dl vs. 159 mg/dl, p = 0.037) were increased in non-diabetic patients. Low-density lipoproteins (LDL) and triglycerides showed no relevant difference in both patients groups (Table 1).

Inflammatory markers such as high sensitive-C-reactive protein (hs-CRP) (p = 0.014), neutrophils (p = 0.002), leukocytes (p = 0.03), and fibrinogen (p = 0.003) were significantly elevated in peripheral blood in diabetic vs. non-diabetic patients. Likewise, HbA_1c_ was higher in diabetics compared to non-diabetics (6.8% vs. 5.8%, p < 0.0001, Table 1).

### NGAL and MMP-9/NGAL serum levels

**Fig. 1:**
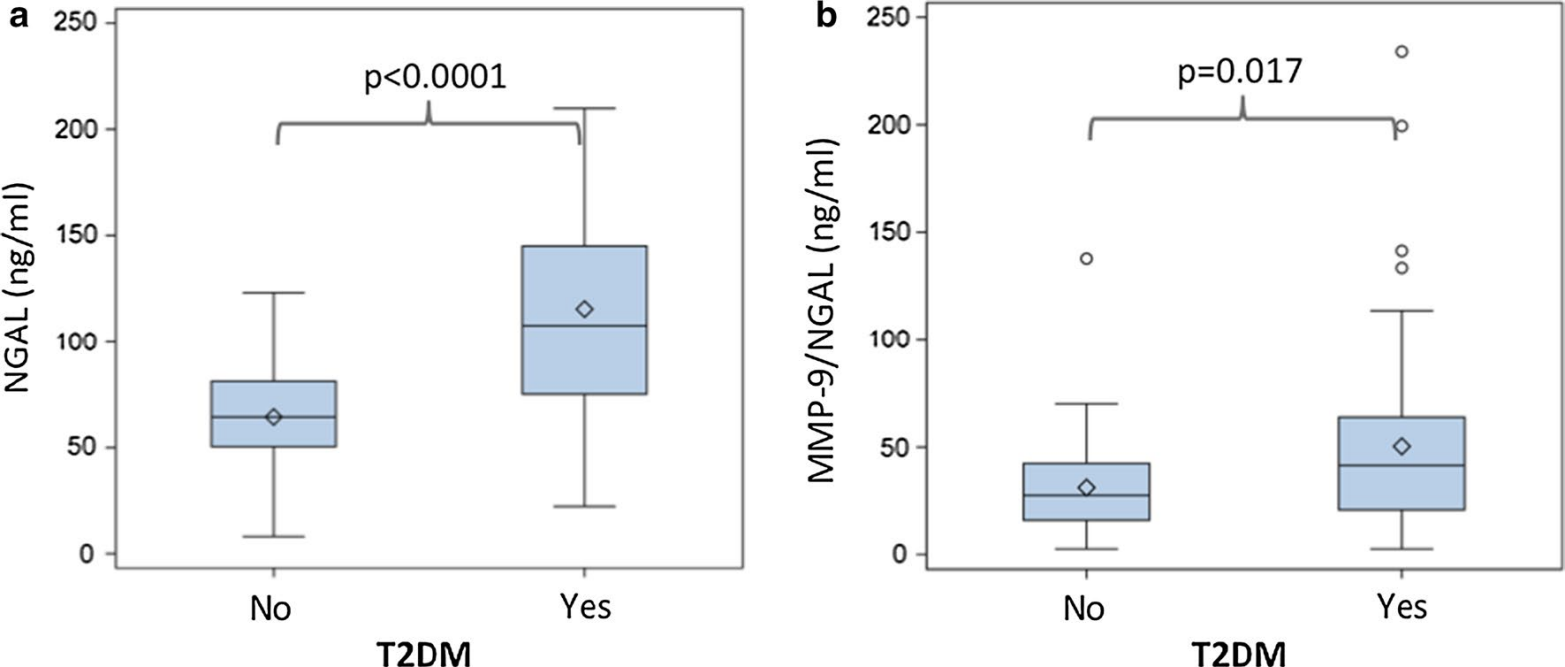
NGAL and MMP-9/NGAL serum levels are higher in diabetics with CAS compared to non-diabetics. Box-whisker plot display serum NGAL (**a**) and MMP-9/NGAL (**b**) concentrations stratified according to the presence of diabetes

Neutrophil gelatinase associated lipocalin was significantly higher in diabetic patients compared to non-diabetics [107.4 ng/ml (75.2–145.0) vs. 64.4 ng/ml (50.4–81.3), p < 0.0001, Fig. 1a]. The same was true for the MMP-9/NGAL complex [41.5 ng/ml (20.8–63.9) vs. 27.6 ng/ml (16.0–42.4), p = 0.017, Fig. 1b].

**Fig. 2:**
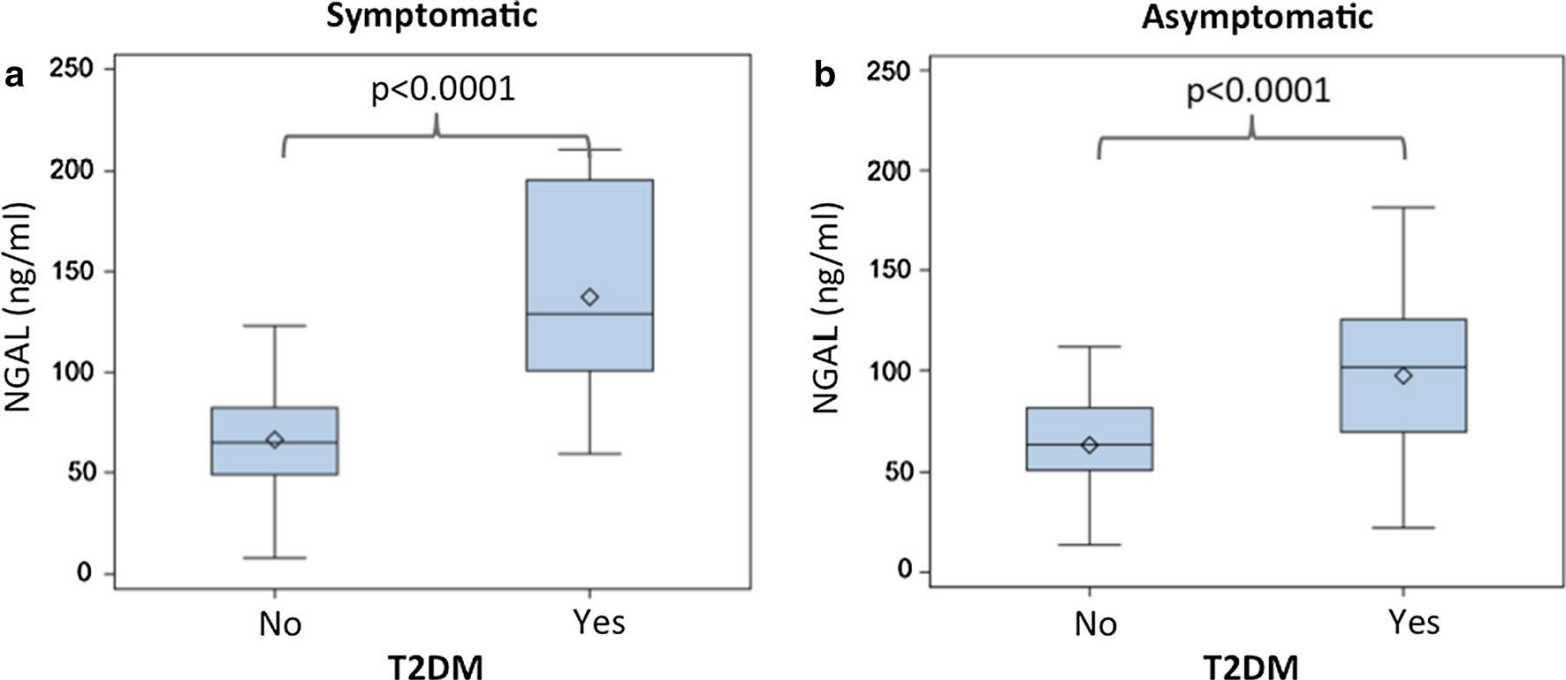
Association of NGAL with the symptomatic presentation of carotid artery stenosis. Box-whisker plots display serum NGAL levels in patients with symptomatic (**a**) and asymptomatic carotid artery stenosis (**b**) stratified according to the presence of diabetes

Type 2 diabetics with symptomatic CAS had significantly higher NGAL levels compared to symptomatic non-diabetics [128.8 ng/ml (100.8–195.6) vs. 64.8 ng/ml (48.9–82.2), p < 0.0001, Fig. 2a]. Likewise, NGAL levels were significantly elevated in asymptomatic patients with T2DM compared to asymptomatic non-diabetics [101.6 ng/ml (70.1–125.3) vs. 63.8 ng/ml (51.0–81.3), p < 0.0001, Fig. 2b]. MMP-9/NGAL levels, however, did not reach statistical significance when comparing symptomatic diabetics [37.7 ng/ml (18.0–60.3)] vs. symptomatic non-diabetics [18.8 ng/ml (16.0–46.7), p = 0.11]. Comparison of MMP-9/NGAL levels between asymptomatic diabetics [45.8 ng/ml (23.2–63.9)] vs. asymptomatic non-diabetics [31.7 ng/ml (17.8–42.4) p = 0.057] revealed a tendency towards higher MMP-9/NGAL levels in diabetics.

HbA_1c_ showed a positive correlation with NGAL levels (r = 0.38, p < 0.001), but not with MMP-9/NGAL levels (r = 0.10, p = 0.23). Positive correlations were also observed between NGAL and creatinine levels (r = 0.29, p = 0.0006) and neutrophil counts (r = 0.26, p = 0.003), respectively. The duration of T2DM did not correlate with NGAL (r = 0.03, p = 0.89) or MMP-9/NGAL levels (r = −0.16, p = 0.42). BMI did not show any correlation with NGAL (r = −0.01, p = 0.95) or MMP-9/NGAL levels (r = 0.17, p = 0.05). Detailed results of Spearman correlation analysis are listed in Table 2.

**Table 2:** Correlation between serum levels of NGAL and MMP-9/NGAL and selected clinical and laboratory parameters

	NGAL (ng/ml)		MMP-9/NGAL (ng/ml)	
	r	p	r	p
HbA_1c_, %	0.38	<0.001	0.10	0.23
Creatinine, mg/dl	0.29	0.0006	−0.012	0.89
Neutrophil count, G/l	0.26	0.003	0.36	<0.0001
Duration T2DM, years	0.03	0.89	−0.16	0.42
BMI, kg/m^2^	−0.01	0.95	0.17	0.05

*r* spearman correlation coefficient, *HbA*_*1c*_ glycated hemoglobin, *BMI* body mass index

### NGAL mRNA expression

Neutrophil gelatinase associated lipocalin mRNA expression was detected at a higher rate in atherosclerotic tissue of patients with T2DM (in 19 of 20 patients, 95%) in comparison to non-diabetics (in 1 of 19 patients, 5%, p < 0.0001). In addition, symptomatic CAS was found more frequently in type 2 diabetics with detectable NGAL mRNA expression compared to non-diabetics [13/19 (68.4%) vs. 1/19 (5.3%), p < 0.0001]. Diabetics with asymptomatic CAS showed a higher prevalence of detectable NGAL mRNA expression in carotid endarterectomy specimens compared to non-diabetics with asymptomatic CAS [6/6 (100%) vs. 0/0 (0%), p < 0.0001].

### Histological findings

Vulnerable, grade VI plaques were found more frequently in patients with T2DM (n = 28/67, 41.79% vs. n = 11/69, 15.94% in non-diabetics, p = 0.0009). Presence of stable, grade VII plaques did not differ significantly between both groups (n = 16/67, 23.88% in diabetics vs. n = 25/69, 36.23% in non-diabetics, p = 0.12), nor did plaques grade V (fibroatheroma) (n = 23/67, 34.33% in diabetics vs. n = 33/69, 47.83% in non-diabetics, p = 0.11). Histological classification according to the AHA revealed only one plaque grade VIII in the non-diabetes group and one plaque grade IV in each of both groups.

### Univariate and multiple linear regression analysis

In the total cohort (both diabetic and non-diabetic patients), symptomatic CAS had an impact towards higher NGAL levels (p = 0.0007) in univariate model. Moreover, histological class VI was associated with higher NGAL levels, whereas grade VII adversely affected NGAL (p < 0.0001). T2DM patients showed significantly higher NGAL levels (p < 0.0001). Additionally, creatinine, neutrophils and plaques ultrasound gave statistically significant results in univariate models with respect to NGAL values. On contrary, BMI did not alter NGAL levels. Multiple linear regression analysis showed the following results: T2DM (p < 0.001), symptomatic presentation (p = 0.023), histological classification (p < 0.0001) and plaque ultrasound (p = 0.004) significantly influence NGAL levels. With respect to MMP9-NGAL levels univariate regression analyses showed significant results for T2DM (p = 0.017), histological classification (p = 0.004), plaque ultrasound (p = 0.0004) and neutrophils (p < 0.0001). Multiple regression models only gave significant results for neutrophils (p = 0.010). Results of both univariate and multiple linear regression analysis are given in detail in Table 3A, B.

**Table 3:** Univariate and multiple linear regression models for serum NGAL (A) and MMP-9/NGAL (B, log-transformed) concentrations in the total cohort

	Univariate models		Multiple model	
	β ± SE^a^	p	β ± SE^a^	p
(A)				
BMI	−0.21 ± 1.01	0.832	0.42 ± 0.67	0.532
Creatinine (rank-transformed)	0.32 ± 0.10	0.002	0.14 ± 0.07	0.056
Neutrophils (log-transformed)	35.9 ± 12.1	0.003	4.4 ± 8.6	0.609
Plaque ultrasound		<0.0001		0.004
“calcified/hard”vs. “soft”	−59.3 ± 7.3		−18.4 ± 7.6	
“mixed” vs. “soft”	−55.5 ± 9.2		−26.1 ± 8.0	
Histological classification		<0.0001		<0.0001
Type VI vs. type V	56.6 ± 7.5		33.0 ± 7.3	
Type VII vs. type V	−21.7 ± 7.4		−18.8 ± 6.8	
Symptomatic (yes vs. no)	27.8 ± 8.0	0.0007	12.9 ± 5.6	0.023
T2DM (yes vs. no)	50.8 ± 6.9	<0.0001	29.1 ± 6.0	<0.0001
(B)				
BMI	0.03 ± 0.02	0.086	0.03 ± 0.02	0.100
Creatinine (rank-transformed)	−0.001 ± 0.0020	0.517	−0.002 ± 0.002	0.391
Neutrophils (log-transformed)	0.89 ± 0.20	<0.0001	0.57 ± 0.22	0.010
Plaque ultrasound		0.0004		0.257
“calcified/hard”vs. “soft”	−0.59 ± 0.15		−0.26 ± 0.19	
“mixed” vs. “soft”	−0.49 ± 0.19		−0.31 ± 0.20	
Histological classification		0.004		0.196
Type VI vs. type V	0.45 ± 0.17		0.27 ± 0.19	
Type VII vs. type V	−0.13 ± 0.17		−0.12 ± 0.17	
Symptomatic (yes vs. no)	−0.08 ± 0.15	0.602	−0.17 ± 0.14	0.239
T2DM (yes vs. no)	0.34 ± 0.14	0.017	0.04 ± 0.15	0.801

^a^Regression parameter (β) ± standard error (SE)

### Metformin treatment

**Fig. 3:**
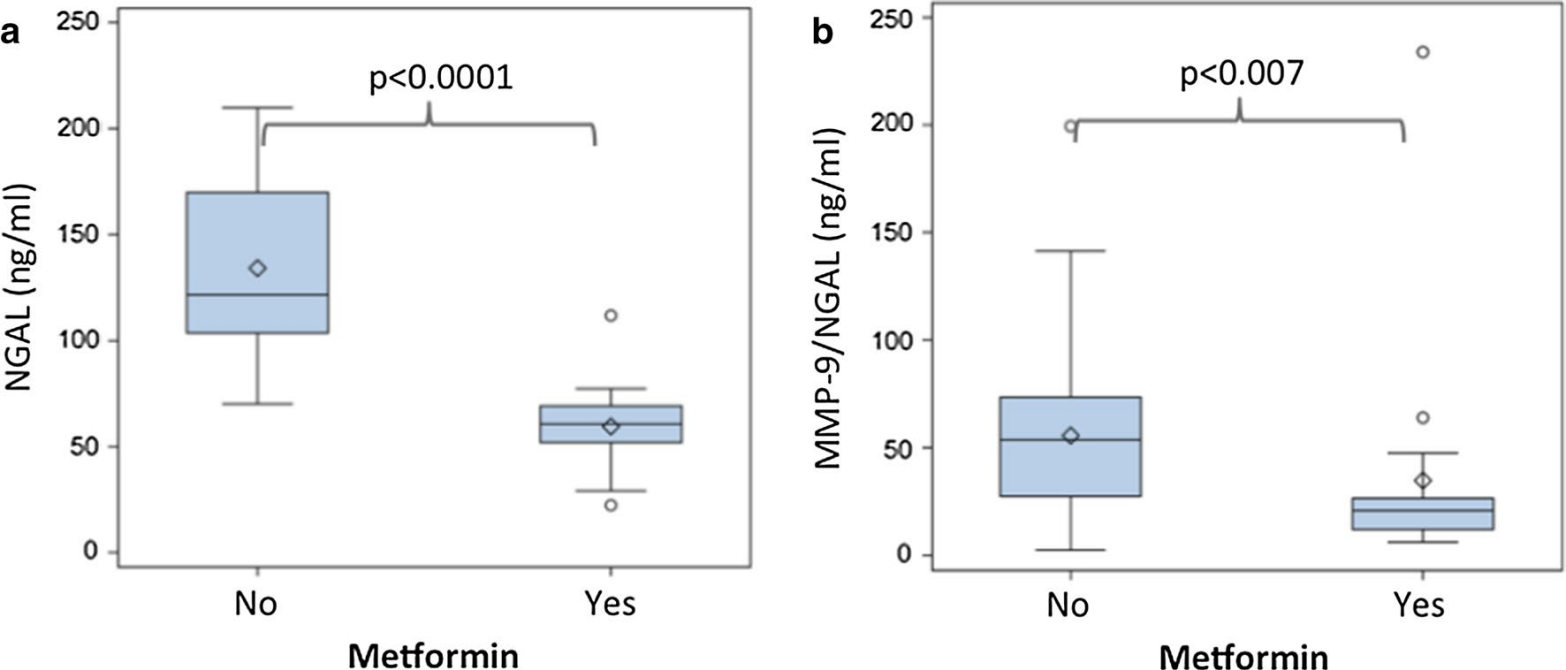
NGAL and MMP-9/NGAL serum levels are lower in metformin-treated patients. Box-whisker plot display serum NGAL (**a**) and MMP-9/NGAL (**b**) concentrations stratified according to metformin treatment

Diabetic patients with metformin therapy (n = 17), regardless of additive treatment with insulin or other anti-diabetic treatment, showed significantly lower NGAL levels [60.7 ng/ml (51.9–69.2) vs. 121.7 ng/ml (103.7–169.9), p < 0.0001, Fig. 3a] as well as MMP-9/NGAL levels [20.8 ng/ml (12.1–26.5) vs. 53.7 ng/ml (27.4–73.4), p = 0.007, Fig. 3b] compared to diabetics without metformin treatment. Insulin treatment alone (n = 16) had no effect on NGAL [88.7 ng/ml (59.0–118.6) vs. 111.6 ng/ml (78.6–145.9), p = 0.14] and MMP-9/NGAL levels [27.5 ng/ml (13.1–66.1) vs. 45.8 ng/ml (24.0–63.0), p = 0.27] compared to diabetics without insulin treatment (n = 51). Likewise, other anti-diabetic medication (n = 18) showed no influence on NGAL [101.2 ng/ml (76.3–126.6) vs. 110.6 ng/ml (72.9–145.9), p = 0.59] and MMP-9/NGAL levels [32.0 ng/ml (18.2–59.1) vs. 44.7 ng/ml (24.0–65.9), p = 0.97] compared to diabetics with insulin and metformin. Within anti-diabetic treatment only metformin significantly influenced NGAL (p < 0.0001) and MMP-9/NGAL levels (p = 0.04) after adjustment in multiple linear regression analyses model. Furthermore HbA_1c_ showed a statistically significant influence on NGAL (p = 0.021) in multiple regression analysis on T2DM patients. Results of both univariate and multiple linear regression analysis are given in detail in Table 4A, B.

**Table 4:** Univariate and multiple linear regression models for serum NGAL (A) and MMP-9/NGAL (B, log-transformed) concentrations in diabetic patients

	Univariate models		Multiple model	
	β ± SE^a^	p	β ± SE^a^	p
(A)				
Insulin	−21.75 ± 14.41	0.136	−10.97 ± 13.30	0.413
Metformin	−74.67 ± 10.98	<0.0001	−74.60 ± 11.84	<0.0001
Other anti-DM medication	−7.56 ± 14.07	0.593	−7.31 ± 11.21	0.517
HBA_1c_	5.81 ± 6.70	0.388	14.12 ± 5.94	0.021
log_Neutrophils	33.68 ± 18.69	0.076	9.57 ± 14.88	0.523
(B)				
Insulin	−0.29 ± 0.27	0.271	1.88 ± 1.02	0.072
Metformin	−0.69 ± 0.247	0.0067	−0.57 ± 0.28	0.043
Other anti-DM medication	0.0087 ± 0.258	0.973	0.04 ± 0.26	0.875
HBA_1c_	0.05 ± 0.123	0.665	0.12 ± 0.14	0.405
log_Neutrophils	0.821 ± 0.339	0.018	0.63 ± 0.35	0.073

^a^Regression parameter (β) ± standard error (SE)

**Fig. 4:**
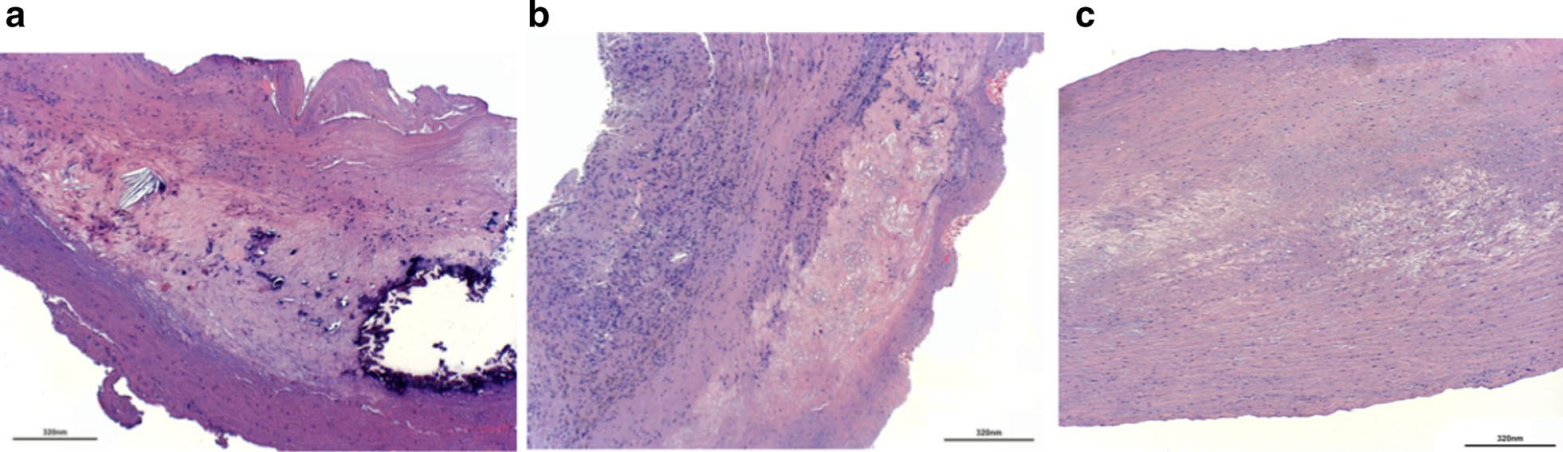
Infiltration of leukocytes in carotid artery lesions is reduced in diabetics under metformin treatment. Light microscopic pictures of human carotid atherosclerotic lesion from non-diabetic patients (**a**), from diabetic patients without metformin intake (**b**) and from diabetic patients with metformin intake (**c**). Hematoxylin–eosin staining. Original magnification ×40. *Scale bar* 320 nm. Representative pictures are shown

Histological analysis of carotid plaques showed moderate signs of inflammation (i.e. infiltration of leukocytes) in non-diabetics (Fig. 4a), whereas in patients with T2DM an infiltration with leukocytes was abundant (Fig. 4b). Finally, histologic analysis showed low signs of inflammation in type 2 diabetics with metformin intake (Fig. 4c) as compared to those without metformin intake (Fig. 4b). Moreover, vulnerable plaques types VI were extremely rare in patients with metformin treatment (n = 2/23, 8.7%).

## Discussion

Inflammatory cells and cytokines trigger inflammatory cascades and play a fundamental role in the development and progression of atherosclerosis [5–12]. In patients suffering from T2DM inflammatory processes are a cause of concern as hyperglycemia and insulin resistance as well as the underlying mechanisms contributing to vascular complications may enhance development of plaque erosion, fissuring and rupture [13, 18, 19, 37]. To our knowledge, this is the first study that elucidates the association between NGAL in blood and carotid plaques and the underlying clinical and histological characteristics in type 2 diabetics with CAS undergoing carotid endarterectomy as well as the influence of metformin treatment on NGAL levels in these patients.

### Serum NGAL and MMP-9/NGAL

In our study, serum NGAL and MMP-9/NGAL levels were significantly elevated, almost twice as high in diabetics compared to non-diabetics. Moreover, multiple linear regression analysis showed a marked impact of T2DM on NGAL levels in our study. However, two previous reports showed contradictory results [38, 39]. Giaginis and coworkers stated that plasma NGAL concentrations did not differ significantly in non-diabetics versus a small group of 33 diabetics with CAS [38]. First of all, the low number of patients may have contributed to these results. Furthermore, this study did not report anti-diabetic treatments. As our present data showed, diabetics under metformin treatment demonstrated significantly reduced serum NGAL and MMP-9/NGAL levels comparable with non-diabetics. Consequently, these factors may have profoundly influenced the sub-group analysis by Giaginis et al. [38]. The second report, which failed to show differences of NGAL levels in patients with T2DM and CAS, was restricted to asymptomatic patients with early CAS. In addition, a relatively high number of young and female patients were included in that study [39]. Consequently, those patient cohorts are not comparable to that analyzed in the present study. Moreover, NGAL mRNA expression was detectable in 95% of patients with T2DM compared to 5% of non-diabetics in our study. This clearly indicates the increased expression of NGAL at cellular level in carotid atherosclerotic lesions in patients with T2DM.

### Pro-inflammatory markers

In our cohort, elevated well-established pro-inflammatory markers and acute phase proteins such as hs-CRP, fibrinogen, and neutrophils, evidenced the inflammatory burden of T2DM compared to non-diabetics. Odegaard et al. [15] reported a positive association between hs-CRP and T2DM in patients from the CARDIA study. Moreover, NGAL has been described as an inflammatory marker, especially in metabolic complications [30, 40]. Two other reports showed a positive correlation between hs-CRP and serum NGAL levels in patients with CAD and in the general population [28, 41]. In addition, plasma NGAL was strongly associated with total leucocyte and neutrophil count [41]. The same was true for the diabetic patients in our study. Additionally we demonstrated here the increased number of leukocytes in carotid plaques of diabetics in comparison to non-diabetics.

HbA_1c_ significantly influenced NGAL, but not MMP-9/NGAL levels in our cohort of patients, which comprised relatively well medically adjusted diabetics with a median HbA_1c_ level of 6.8%. Moreover, besides MMP-9, NGAL possesses various ligands such as siderophores contributing to the broad range of different functions [42]. The latter may explain why in case of NGAL elevation MMP-9/NGAL complex has not to be necessarily elevated, too. The duration of T2DM had no effect on NGAL levels, illustrating that the current inflammatory state along with the current HbA_1c_ level is of more importance. These findings are in agreement with previous reports [38, 39].

### Risk for cerebral embolization

CAS in type 2 diabetics is characterized by an increased risk for cerebral embolization. In the present study proven embolization was almost twice as high in patients with T2DM as compared to non-diabetics, which confirmed previous results [19, 43]. Interestingly, NGAL levels were highest in diabetic patients with symptomatic CAS. Moreover, NGAL mRNA expression was found in atherosclerotic tissue of 95% of patients with T2DM compared to 5% in non-diabetics. Out of these patients, those with detectable mRNA expression for NGAL were more prone towards embolization. Although comparable data in patients with T2DM are missing, these findings are supported by Boeckhorst et al., who reported that tissue NGAL expression was elevated in case of intra-plaques hemorrhage or luminal thrombus [27]. In addition, carotid plaque NGAL was associated with high numbers of macrophages and high IL-6 and IL-8 levels [27]. These findings are in line with our previous investigation [23]. In addition, various studies demonstrated higher plaque MMP-9 levels in type 2 diabetics compared to non-diabetics [16]. Moreover, plaque ulceration was reported to be significantly more common in carotid plaques of a cohort of 30 diabetics [19]. Likewise, type VI plaques were seen more frequently in patients with T2DM in our study. In addition, multiple linear regression analysis showed a significant influence of T2DM, symptomatic presentation of carotid stenosis and grade VI plaques on NGAL levels. In contrast, advanced grade of CAS did not alter plasma NGAL levels, as reported by Giaginis et al. [38]. Although it is far too early to describe serum NGAL as reliable biomarker for vulnerable CAS in patients with T2DM, enhanced NGAL concentrations in the circulation did not meet statistical significance for patients with a history of peripheral artery disease or CAD as described by Giaginis et al. previously [38].

### Effect of metformin

Finally, we investigated the association of NGAL levels with metformin treatment that is generally prescribed as the first line treatment of T2DM. Next to glucose lowering effect, metformin was recently discussed to have anti-inflammatory and other pleiotropic effects [44, 45]. Xu et al. showed, that metformin lowered hs-CRP, IL-6, and TNF-α levels in diabetics and concluded that metformin has a protective potential to ameliorate the pro-inflammatory response in patients with CAS [44]. Moreover, metformin reduced endothelial cell production of IL-6 in vitro [46] and below-the-knee arterial calcification score in type 2 diabetics [47]. These patho-mechanisms may contribute to stabilization of carotid plaques in patients with T2DM and improve all-cause mortality and cardiovascular events [48]. An extremely low number of grade VI plaques in patients with T2DM and metformin intake in our study add to these considerations.

### Strength of the study

The strength of our investigation is a detailed work-up of histological specimen according to the AHA classification against the background of the clinical situation. In addition, NGAL mRNA analysis was performed to gain information about NGAL expression in the atherosclerotic lesion directly. We analyzed for the first time the association of metformin and NGAL levels in diabetic patients with CAS.

### Limitations of the study

On the other hand the present study has several limitations. The study was not designed with three arms analyzing type 2 diabetics without metformin compared to those with metformin and non-diabetics in three groups of equal number of patients. However, post hoc analysis of patients with T2DM revealed the profound anti-inflammatory effect of metformin on pro-inflammatory protein NGAL. In addition, the number of patients included in the investigation was limited. As a consequence, further studies are necessary to rule out the exact pathomechanisms of metformin on NGAL and MMP-9/NGAL levels in a larger series of patients.

## Conclusion

In summary, the present investigation showed that type 2 diabetics with high-grade CAS undergoing carotid endarterectomy have increased levels of NGAL systemically as well as locally within the plaque as compared to non-diabetics. Even in asymptomatic patients NGAL was elevated in diabetics compared to non-diabetics. NGAL mRNA carotid plaque expression was abundant in diabetics and rarely found in non-diabetics. NGAL levels being significantly influenced by presence of T2DM, HbA_1c_ and vulnerable plaques may indicate dangerous lesions prone for embolization. These findings reflect the fact that proven embolization was almost twice as frequent in type 2 diabetics, which highlights the need for aggressive therapy in these patients. Metformin was obviously able to reduce the increased inflammatory state by lowering NGAL serum levels as well as leukocytes infiltration in carotid plaques.

## Abbreviations

AHA: American Heart Association
BMI: body mass index
CAD: coronary artery disease
CAS: carotid artery stenosis
CRP: c-reactive protein
H&E: hematoxylin and eosins
HbA_1c_: glycated hemoglobin
HDL: high-density lipoproteins
IL: interleukin
LDL: low-density lipoproteins
MMP-9: matrix metalloproteinase-9
NGAL: neutrophil gelatinase associated lipocalin
T2DM: type 2 diabetes mellitus
TIA: transient ischemic attacks
